# Molecular evidence for the first records of facultative parthenogenesis in elapid snakes

**DOI:** 10.1098/rsos.171901

**Published:** 2018-02-21

**Authors:** L. Allen, K. L. Sanders, V. A. Thomson

**Affiliations:** 1 Venom Supplies, PO Box 547, Tanunda, South Australia 5352, Australia; 2 School of Biological Sciences, and the Environment Institute, The University of Adelaide, Adelaide, South Australia 5005, Australia

**Keywords:** snake, Elapidae, facultative parthenogenesis, reproduction, ddRAD-seq, parentage

## Abstract

Parthenogenesis is a form of asexual reproduction by which embryos develop from unfertilized eggs. Parthenogenesis occurs in reptiles; however, it is not yet known to occur in the widespread elapid snakes (Elapidae), which include well-known taxa such as cobras, mambas, taipans and sea snakes. Here, we describe the production of viable parthenogens in two species of Australo-Papuan elapids with divergent reproductive modes: the oviparous coastal/Papuan taipan (*Oxyuranus scutellatus*) and the viviparous southern death adder (*Acanthophis antarcticus*). Analyses of nuclear SNP data excluded paternity for putative fathers and convincingly demonstrated asexual reproduction, thus representing the first evidence of facultative parthenogenesis in Elapidae. Our finding has broad implications for understanding the evolution of reproductive diversity in snakes, as well as managing the conservation of genetic diversity in wild and captive populations.

## 1. Introduction

Parthenogenesis is an asexual mode of reproduction by which embryos develop from unfertilized eggs. The importance of this reproductive mode in vertebrates is now widely recognized, with observations of parthenogenesis in several classes, including reptiles [1]. In snakes, there is evidence of two naturally occurring modes of asexual reproduction. Obligatory parthenogenesis (OP) is found in exclusively parthenogenic species such as the Brahminy Blind Snake (*Indotyphlops braminus*) which have all-female populations [2]. Facultative parthenogenesis (FP) appears to be more common and is found in snakes that reproduce sexually as well as asexually and thus have male/female populations [3]. FP in vertebrates was considered a probable artefact of captivity until evidence was reported from wild populations, including two species of pitviper snakes (Viperidae) [4]. A second generation of parthenogens has also been confirmed in another vertebrate (captive white spotted bamboo shark: [5]), but this has yet to be reported in snakes.

The process of FP occurs via ‘automixis’ and is attributed to terminal fusion, whereby the egg nucleus fuses with a second polar body and diploidy is restored [6]. However, Booth & Schuett [3] note that without confirmation of heterozygosity, gamete duplication in unfertilized eggs cannot be rejected as an alternative mechanism of parthenogen production. Records of FP in ‘advanced’ snakes (caenophidians + colubroids) indicate that fertility rates of litters/clutches containing parthenogens are low, often contain stillborns, and only male parthenogenetic offspring are produced as a result of ZW sex chromosome systems (female heterogamety) in these specie [3,7–9]. By contrast, alethinophidians (Boidae and Pythonidae) produce only female parthenogens as a result of XY (male heterogamety). In both cases, the progeny are half clones of the mother. Despite a growing list of snake taxa for which we have genetic confirmation of FP (see [10]), none have been reported from the widespread and species-rich Elapidae, which includes well-known medically significant species such as cobras, mambas, taipans and sea snakes.

Here, we present genetic evidence of FP in two species of elapids with different reproductive modes: the oviparous Papuan/coastal taipan (*Oxyuranus scutellatus*) and viviparous southern death adder (*Acanthophis antarcticus*). The reproductive accounts resulting in parthenogens were atypical to our previous reproductive record for the species, and followed characteristics outlined by Booth & Schuett [9,11]. Females had not been in contact with males for extended periods, fertility rates of clutches/litters were very low (table 1), all resulting offspring were male and some stillborn/developmental abnormalities were evident.

**Table 1.** Reproductive output and health status of parthenogens from female Papuan/coastal taipans (*O. scutellatus*) and southern death adder (*A. antarcticus*).

species	individual	fertile	infertile	development	physical appearance
coastal taipan (*O. scutellatus*)	mother #1	2	14	both normal	one parthenogen appears normal, the second having malformed scales
coastal taipan (*O. scutellatus*)	mother #2	1	7	normal	1 deformed eye—otherwise normal
Papuan taipan (*O. scutellatus*)	mother #3	1	10	normal	normal
southern death adder (*A. antarcticus*)	mother #4	4 (2 live, 2 stillborn)	9	slow, and died <2 months after birth	normal

The coastal/Papuan taipan occurs in subtropical to tropical coastal regions of Australia and southern PNG. The southern death adder occurs in temperate to tropical regions of southern and eastern Australia. Both species are large bodied, highly venomous and medically significant. Reviewed data from published reproductive accounts of the elapids show the southern death adder produces mean 17.6 (*n* = 80) neonates and the taipan produces mean 14 (*n* = 67) [12]. The taipan is also known to reproduce from stored sperm [13,14]. Taipans from PNG were long considered a subspecies of the coastal taipan after Slater [15] but have since been shown to be synonymous [10]. Although now synonymous given the significance of geographic relief between the two populations, we refer to the ‘Papuan/coastal taipan (*O. scutellatus*)’ throughout this manuscript.

We used double-digest restriction site-associated DNA (ddRAD-seq) nuclear SNP markers to genotype reproductive females, their offspring and captive males that potentially sired these offspring. Our findings represent the first molecular verification of FP in the Elapidae and thus extend the current knowledge of FP in snakes.

## 2. Material and methods

### 2.1. Snake maintenance and breeding

Females #1 and #2 were multi-generation captive coastal taipan bred and raised at our facility, whereas female #3 was a Papuan taipan wild collected as an adult from Saibai Island in the Torres Strait. The female southern death adder was wild collected from the Eyre Peninsula, South Australia. Snakes were maintained individually with a thermostatically controlled heat source which provides a temperature gradient allowing for thermoregulation. Death adders were exposed to thermal seasonal shifts where the heat source was reduced gradually over the cooler months and slowly raised leading into warmer months simulating natural temperate environments. At appropriate times of year, male snakes were introduced to female enclosures for 1–5 day periods for potential mating. Females were noticeably gravid when they became larger in their posterior region, restless and refused food late in gestation.

Upon parturition, neonatal death adders were removed from the mothers and housed individually. The Papuan/coastal taipan eggs were removed from the mother's enclosure upon deposition and artificially incubated.

### 2.2. Genotyping and analyses

Tissue samples from adults and offspring were collected, and DNA was extracted using a ‘salting-out’ method [16] and the extracts quantified using the Quantus Fluorometer (Promega) as per the manufacturer's instructions. Double-digest restriction-associated DNA (ddRAD-seq) libraries were made in batches of 96 including a library blank control following the protocol of Poland *et al*. [17] with some modifications. Two hundred nanograms of DNA were digested at 37°C for 2 h using 8 U of *PstI* (six-base recognition site, CTGCAG) and *HpaII* (four-base recognition site, CCGG) in 20 µl of 1× CutSmart Buffer (New England Biosciences (NEB)).

Uniquely barcoded adapters were then ligated to the DNA in 40 µl volumes, consisting of 20 µl of digested DNA, 200 U of T4 ligase, 0.1 pmol of forward (rare) and 15 pmol of reverse (common) adapters and 1× T4 Buffer. The mixture was incubated at room temperature for 2 h, and then heat killed at 65°C for 20 min. Ligation products were pooled into four pools of 24 samples each. Pooled libraries were purified using the QIAquick PCR purification kit (Qiagen) and eluted in 120 µl of EB buffer (Qiagen).

PCR reactions to add the full-length Illumina adapters [17] were performed in eight replicates per library pool in 30 µl volumes containing 10 µl of purified library, 1× Hot Start Taq Master Mix (NEB) and 0.66 µM each of the forward and reverse primers. The PCR conditions were: 95°C for 30 s, 16 cycles of 95°C for 30 s, 65°C for 20 s and 68°C for 30 s, followed by 68°C for 5 min and 25°C for 1 min. The eight replicates per library were re-pooled and purified as above, eluting in 30 µl of EB buffer (Qiagen). We then used a two-step double-SPRI protocol [18] to pool all four of the libraries and to select for fragments between 100 and 300 bp using a homemade SPRI bead mix [19]. Pooled libraries were sequenced in 1 × 75 bp (single-end) high output reactions on the Illumina Next-seq at the Australian Genome Research Facility, Adelaide.

#### 2.2.1. Sequence processing

We used STACKS v. 1.47 pipeline [20,21] to process the sequence data for each species separately, employing parameters previously identified as maximizing matching SNP calls and minimizing mismatching SNP calls between sample replicates. Raw sequencing reads were demultiplexed using GBSX [22]. These demultiplexed reads were chastity filtered, had regions with quality less than Q10 trimmed from either end of the read, had cut-sites removed, had adapters removed (allowing for one mismatch) and truncated to 57 bp using the BBTools modules (bbduk.sh and bbduk2.sh; [23]). Samples with more than 500 000 reads per sample were considered well sequenced and continued in the analysis. RAD loci were identified for each sample using the *ustacks* module, requiring a minimum stack read depth of five (*m* = 5) and a maximum of one nucleotide mismatches (*M* = 1) between stacks at a locus. Loci with more than 50 stacks (mls = 50) and more reads than two standard deviations above the mean were filtered as they may map to multiple points on the genome. A ‘deleveraging algorithm’ was used to try to resolve over-merged loci. A catalogue of consensus loci among individuals for each species was constructed with the *cstacks* module using the *ustacks* output files. Loci were recognized as homologous across individuals if they mismatched at four or fewer bases (*n* = 5). Alleles were identified in each individual against this catalogue using the module *sstacks*. The module *populations* was used to remove potential homologues by filtering out loci with heterozygosity greater than 0.8. Loci with a minimum minor allele frequency less than 0.05 were filtered out, as were loci not sequenced in all samples within a species and the resulting SNP datasets were output to a PLINK format file (i.e. ped and map files).

The R package Rhh [24] was used to test three measures of individual multilocus heterozygosity: internal relatedness (IR; [25]), standardized heterozygosity (SH; [26]) and homozygosity by loci (HL; [27]). IR can be used to infer inbreeding from multilocus heterozygosity data using allele frequencies; however, it underestimates heterozygosity of individuals carrying rare alleles. Therefore, HL is also used as it weighs the contribution of each locus to the homozygosity index depending on their allelic variability. SH takes into account that not all samples will be genotyped for all of the same loci by ensuring that the heterozygosity of all individuals is measured on the same scale. Standardized individual heterozygosity (SH) is calculated such that SH = proportion of heterozygous typed loci/mean heterozygosity of typed loci [26]. Parthenogens are expected to have positive IR values, low SH values and high HL values [24].

## 3. Results and discussion

**Figure 1.**
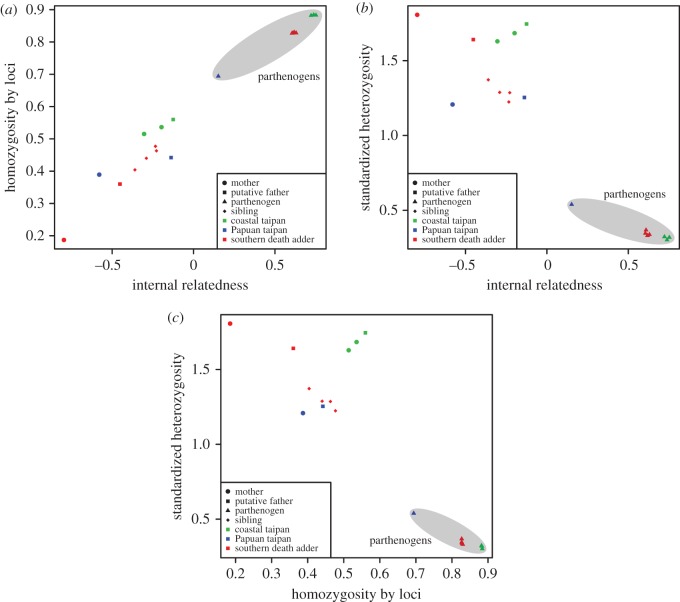
Plots showing relationships between relatedness and heterozygosity/homozygosity measures. (*a*) Relationship between IR and SH; (*b*) relationship between IR and HL; (*c*) relationship between SH and HL. The shape of the symbols represents the parent/offspring and the colour represents the taxa.

Our study robustly demonstrates multiple cases of FP resulting in viable male offspring in two species of elapids with different reproductive modes: the oviparous Papuan/coastal taipan and the viviparous southern death adder. Booth & Schuett [9] documented long-term sperm storage of approximately 5 years followed by the production of viable offspring in an eastern diamondback rattlesnake (*Crotalus adamanteus*). All the females in our study had reproduced sexually with males prior to producing the parthenogens reported here. Hence, it was necessary to exclude the possibility that offspring were produced using sperm stored from these previous breeding events (i.e. eliminating all sampled males as sires was not sufficient evidence for FP). Molecular analysis of the Papuan/coastal taipan and southern death adder putative parthenogen samples yielded genotypes that had high IR (greater than 0.152), i.e. significantly greater than zero (table 2 and figure 1*a,b*), which is indicative of parthenogenesis [24]. The SH was also low (0.302–0.539) and HL was high (0.694–0.884) in the putative parthenogens, also consistent with parthenogenesis (table 2 and figure 1*c*).

**Table 2.** Estimates of parental and descendent similarity, using IR, SH and HL. Parthenogens are expected to have a positive IR, *p-value of less than 0.05 for *t*-test of IR values for the group of parthenogens (or genetic siblings) greater than 0. Parthenogens are expected to have low SH and high HL. All *p*-values have been corrected using the Bonferroni corrections. The Papuan taipan dataset and the parthenogen 2–1 of the coastal taipan have been excluded from all *t*-tests as there is only one parthenogen and *t*-tests require more than one observation to be tested. Parthenogens are highlighted in bold.

species	individual	internal relatedness (IR)	standardized heterozygosity (SH)	homozygosity by loci (HL)
coastal taipan (*O. scutellatus*) loci = 3715	mother #1	−0.299	1.628	0.516
	**parthenogen 1–1**	**0**.722*	**0**.**323**	**0**.**882**
	**parthenogen 1–2**	**0**.738*	**0**.**302**	**0**.**884**
	mother #2	−0.194	1.683	0.537
	**parthenogen 2–1**	**0**.**752**	**0**.**319**	**0**.**883**
	putative father	−0.125	1.745	0.560
Papuan taipan (*O. scutellatus*) loci = 2462	mother #3	−0.575	1.207	0.389
	**parthenogen 3–1**	**0**.**152**	**0**.**539**	**0**.**694**
	putative father	−0.138	1.254	0.442
southern death adder (*A. antarcticus*) loci = 1311	mother #4	−0.792	1.806	0.187
	**parthenogen 4–1**	**0**.630*	**0**.**338**	**0**.**828**
	**parthenogen 4–2**	**0**.605*	**0**.**346**	**0**.**827**
	**parthenogen 4–3**	**0**.618*	**0**.**331**	**0**.**830**
	**parthenogen 4–4**	**0**.609*	**0**.**368**	**0**.**827**
	genetic sibling 1	−0.290	1.288	0.440
	genetic sibling 2	−0.234	1.224	0.477
	genetic sibling 3	−0.360	1.372	0.404
	genetic sibling 4	−0.228	1.286	0.463
	putative father	−0.452	1.641	0.360

The clutches and litter of parthenogens reported here add to other documented accounts of parthenogenetic reproduction in the caenophidians, yielding low fertility rates (i.e. low fertile versus infertile clutch ratio) and phenotypically only males [3,8,9]. Sex of parthenogen offspring was determined to be male by way of probing and manually everting the hemipenis. We were unable to confirm the sex of the parthenogens genetically as the structure and content of the Z and W sex chromosomes are largely unknown from the few snake genomes that exist, so we could not confirm the lack of female parthenogens by mapping the ddRAD-seq data to the published snake genomes. Nor did we genotype enough confidently sexed males and females to definitively identify sex-specific ddRAD markers [28]. With regard to the health of the parthenogens, the litter of death adders included stillborn offspring that exhibited post-cranial developmental abnormalities like those reported in the checkered garter snake (*Thamnophis marcianus*) by Booth & Schuett [3]. The apparent health of our parthenogens varies individually. All Papuan/coastal taipan parthenogens remained alive at the time of publication with the oldest of which being approximately 3.5 years old and the two death adders failed to thrive dying less than 2 months after birth. Two of the four Papuan/coastal taipans appear robust and as normal sexually produced specimens would, with the other two displaying slight physical abnormalities (table 1). Irrespective of this all Papuan/coastal taipans appear to be robust and developing normally.

Our findings represent the first evidence of FP in the Elapidae, which extends our knowledge of FP in snakes. More targeted research is required to determine the prevalence of this alternative reproductive mode in the elapids, particularly the taxonomic breadth and ecological role of its occurrence in natural populations. Better understanding of the effects of parthenogenetic reproduction on heterozygosity and fertility over multiple generations is also needed to inform conservation of genetic diversity in wild and captive snake populations.

## Data accessibility

Data available from the Dryad Digital Repository: https://dx.doi.org/10.5061/dryad.dq40b [29].
